# Risk factors for conversion to mastectomy due to positive margins in early breast cancer patients when choosing breast-conserving surgery: a retrospective cohort study

**DOI:** 10.3389/fonc.2026.1835556

**Published:** 2026-06-11

**Authors:** Ning-chen Wang, Xing Zhang, Yuan-yuan Xu

**Affiliations:** 1 Nursing Department, The Affiliated People’s Hospital of Ningbo University, Ningbo, Zhejiang, China; 2 Thyroid and Breast Surgery Department, The Affiliated People’s Hospital of Ningbo University, Ningbo, Zhejiang, China; 3 Neurosurgical Department, The Affiliated People’s Hospital of Ningbo University, Ningbo, Zhejiang, China

**Keywords:** breast-conserving surgery, conversion to mastectomy, early breast cancer, positive surgical margins, risk factors

## Abstract

**Background:**

Breast-conserving surgery (BCS) offers oncologic safety and a superior quality of life for patients with early breast cancer (BC). However, conversion to mastectomy (CMT) owing to positive margins remains unavoidable in some cases. This study explored the risk factors contributing to this issue.

**Methods:**

This retrospective study analyzed the clinical records of patients who underwent BC surgery between January 1, 2015, and December 31, 2024. Participants were stratified into CMT and BCS groups according to the surgery they received. Demographic and clinical variables were compared between the cohorts. Potential risk factors were assessed using univariable and multivariable logistic regression, and receiver operating characteristic (ROC) curve analysis was used to assess predictive performance.

**Results:**

Among 290 patients (31 in the CMT group and 259 in the BCS group), five parameters demonstrated significant association with CMT in univariable analysis: body mass index (BMI), multifocality, tumor size, false-negative intraoperative frozen section margin, and ductal carcinoma *in situ* (DCIS) component (*P* < 0.05). Multivariable logistic regression analysis established four robust predictors of CMT: low BMI (odds ratio [OR] 4.611, *P* = 0.001), multifocality (OR 4.863, *P* = 0.026), larger tumor size (OR 3.197, *P* < 0.001), and DCIS component (OR 5.308, *P* = 0.035).

**Conclusions:**

Low BMI, multifocality, larger tumor size, and DCIS components independently predicted CMT in patients undergoing BCS. Although these results should be interpreted with caution, identifying these factors preoperatively may enhance surgical planning and patient outcomes.

## 1 Introduction

Breast cancer (BC) is one of the most common malignant tumors among women worldwide (1). In 2020, BC ranked first in incidence and fifth in mortality with an estimated 2.3 million new cases and 650,000 deaths globally (2, 3). Surgical management of BC primarily includes breast-conserving surgery (BCS) and mastectomy. Compared with mastectomy, BCS combined with adjuvant radiotherapy has become a standard treatment approach, offering comparable survival outcomes and better cosmetic results. Currently, approximately 60-70% of patients diagnosed with BC undergo BCS (4, 5). The aim of BCS is to achieve complete tumor removal while preserving breast appearance. Obtaining tumor-free (negative) surgical margins is essential to minimize the risk of local recurrence (6). If negative margins are not obtained, re-excision is generally performed to remove residual tumor tissue; however, conversion to mastectomy (CMT) becomes inevitable in patients with repeated positive margins. The reported re-excision rate following BCS ranges from 18% to 56%, with 21.5% of these patients ultimately requiring mastectomy (6–8). This unplanned CMT leads to poor cosmetic outcomes, increased complication rates, and a greater healthcare burden.

Previous studies have recommended widening the excision margin to lower the risk of positive margins, thereby decreasing the likelihood of re-excision among high-risk patients undergoing BCS. Some studies have focused on the predictive factors for positive margins (9–11). However, to the best of our knowledge, no study has specifically investigated the risk factors for CMT due to positive margins in patients with BC initially selected for BCS.

## 2 Methods

### 2.1 Study design and eligibility

This retrospective study was conducted at the Affiliated People’s Hospital of Ningbo University. The hospital’s electronic medical records were reviewed to identify patients who underwent BC surgery between January 1, 2015, and December 31, 2024. The inclusion criteria for this study were patients aged ≥ 18 years who underwent BCS. The exclusion criteria included stage III-IV BC, male sex, bilateral BC, previous breast irradiation, or receipt of neoadjuvant therapy. This study was conducted in accordance with the principles of the Declaration of Helsinki of the World Medical Association and was approved by the Ethics Committee of the Affiliated People’s Hospital of Ningbo University (2025-082). The requirement for written informed consent was waived due to the study’s retrospective design. To protect patient privacy, all data were anonymized before use, and relevant prescribed guidelines were implemented in this study.

### 2.2 Surgical procedure of the breast

Each patient diagnosed with BC underwent preoperative ultrasound-guided tumor localization with a skin mark and was placed under general anesthesia in the supine position, with the affected upper limb abducted and a soft pillow placed under the shoulder. The surgical area was disinfected and draped routinely. The incision site was determined based on tumor location, and the skin and subcutaneous fascia were incised accordingly. Excision was performed to include at least 1 cm of normal mammary tissue surrounding the tumor, including the pectoral fascia. Six tissue specimens, each less than 0.5 cm thick, were excised from the cavity walls and labeled as superior, inferior, medial, lateral, basal, and subcutaneous margins. Frozen section analysis of the cavity-shaved margins was performed immediately by a pathologist. Negative margins were defined as the absence of invasive carcinoma or ductal carcinoma *in situ* (DCIS) component in the cavity-shaved margins. Positive margins were re-excised, and the new margins were analyzed using intraoperative frozen sections. If the same margin remained positive twice or reshaping the breast was technically difficult after tumor removal, CMT was performed immediately, with or without reconstruction. All samples were embedded in paraffin for permanent pathological examination. If a positive margin was detected on permanent pathology, reoperation was performed after BCS. Whether undergoing re-excision or direct CMT, patients made the surgical decision after being fully informed of the associated risks and benefits. The final determination of successful BCS was based solely on negative margins on permanent pathology.

Patients in the BCS group underwent tumor resection with placement of four clips in the tumor bed (for boost radiotherapy (RT) planning) and simple surgical closure of the wound. No further reconstructive procedures were performed.

### 2.3 Surgical procedure of the axillary lymph nodes

Sentinel lymph node biopsy was performed in patients with no suspicious axillary lymph nodes on preoperative imaging and physical examination. Otherwise, a preoperative core needle biopsy was performed, and an axillary lymph node dissection was performed when the lymph nodes were involved. During surgery, the sentinel lymph nodes were sent for frozen section analysis. If the number of metastatic axillary lymph nodes was less than or equal to two, further axillary dissection was omitted.

### 2.4 Postoperative treatment

Patients with BC received individualized adjuvant therapies, including chemotherapy, radiotherapy, targeted therapy, endocrine therapy, and immunotherapy, based on tumor histology and the type of surgery performed.

### 2.5 Follow-up regimen

Patients were followed up every three months during the first two years post-surgery, every six months from two to five years, and annually for more than five years post-surgery. Additional examinations were conducted promptly if any abnormalities were detected. Follow-up assessments included physical examination, laboratory tests, and imaging studies.

### 2.6 Data collection

Based on the final surgical approach, patients who successfully underwent BCS were classified into the BCS group, and those who underwent re-excision by mastectomy were included in the CMT group. The following data were collected: demographic characteristics; menopausal status; tumor size; tumor location; clinical stage; histological type; tumor grade; clinical subtype (based on immunohistochemistry); multifocality; estrogen receptor status; progesterone receptor status; human epidermal growth factor receptor status; lymphovascular invasion; and axillary lymph node metastasis. For a multifocal tumor within the same breast, only the largest lesion was included in the analysis of tumor characteristics.

### 2.7 Statistical analysis

Continuous variables were summarized as mean ± standard deviation and compared using independent t-tests or the Mann-Whitney U test, depending on data distribution. Categorical variables were assessed using the chi-square test or Fisher’s exact test, as appropriate. To determine risk factors for conversion, univariable exact logistic regression was first performed. Variables showing a trend toward significance (*P* < 0.10) were entered into a multivariable logistic regression model. The predictive performance of the final logistic model was evaluated using receiver operating characteristic (ROC) curve analysis, which also determined optimal cutoff values for the identified risk factors. All statistical analyses were performed using SPSS (version 22.0; IBM Corp., Armonk, NY, USA), and statistical significance was defined as *P* < 0.05.

## 3 Results

**Figure 1:**
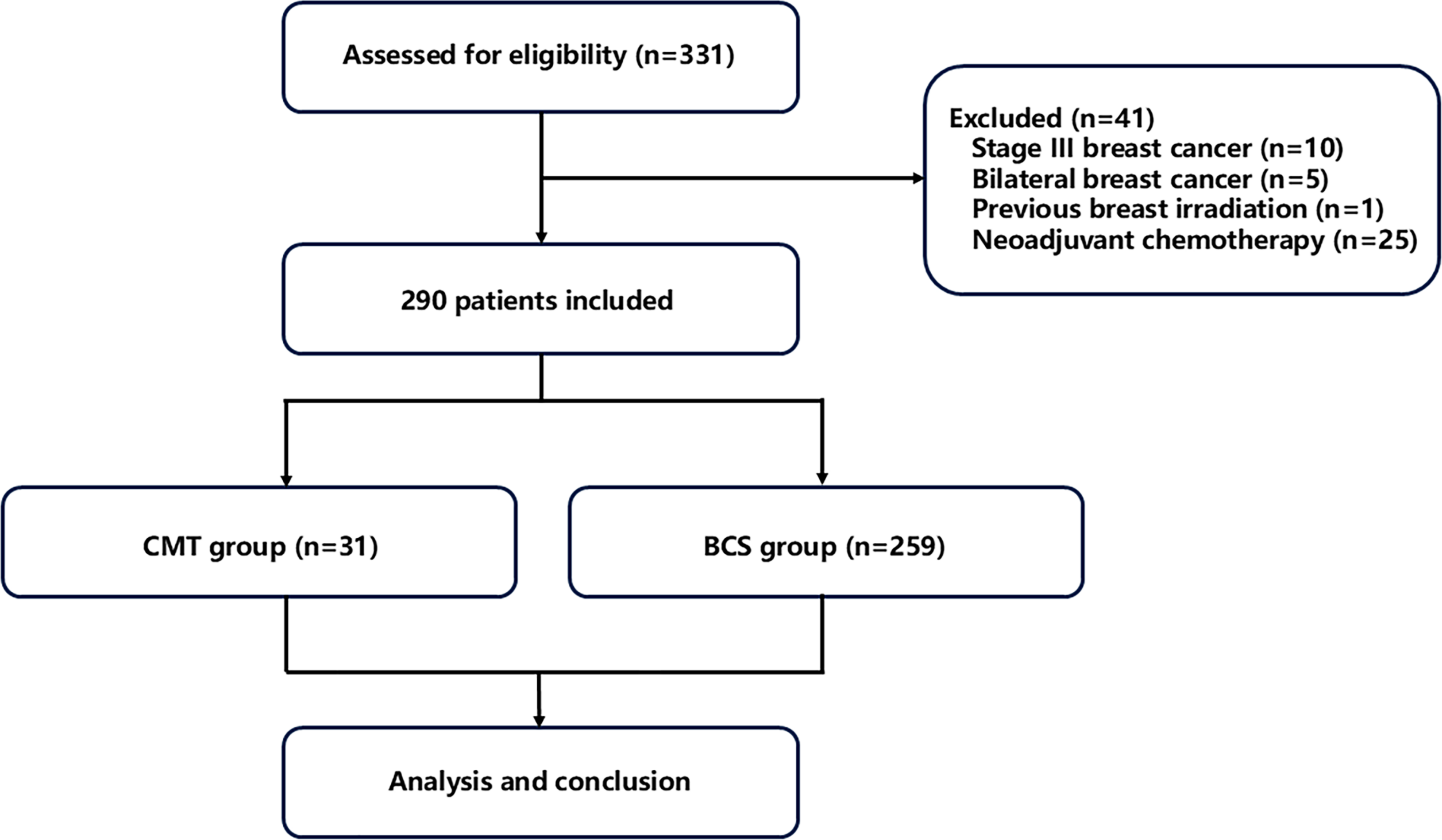
Patient selection process flowchart. CMT, conversion to mastectomy; BCS, breast-conserving surgery.

Altogether, 290 female patients were included in this study, with a mean age of 53.1 ± 12.2 years. The BCS and CMT groups comprised 259 and 31 patients, respectively. In this cohort, 10.7% of patients underwent CMT and nine patients (3.1%) received secondary surgery due to positive margins identified on permanent pathology, which were inconsistent with intraoperative negative margins determined by frozen section analysis. **Figure 1** illustrates the study algorithm and analysis process.

Demographic and clinical characteristics were compared between the two groups. No significant differences were observed in age, tumor palpability, postmenopausal status, tumor location, preoperative magnetic resonance imaging, clinical stage, histological grade, clinical subtype, Ki-67 index, lymphovascular invasion, or axillary lymph node metastasis (*P* > 0.05). Significant differences were observed in body mass index (BMI), multifocality, tumor size, false-negative margins on intraoperative frozen sections, and histological type (*P* < 0.05) (**Table 1**).

**Table 1:** Demography and clinical characteristics of patients between the two groups.

Variables	CMT group (n = 31)	BCS group (n = 259)	*P* value
Age (years)	51.0 ± 11.4	53.4 ± 12.2	0.293
BMI	21.8 ± 1.2	24.0 ± 2.7	<0.001
Palpability (n,%)	4 (12.9%)	43 (16.6%)	0.597
Multifocality (n,%)	6 (19.4%)	12 (4.6%)	0.001
Post-menopause (n,%)	11 (35.5%)	138 (53.3%)	0.061
Tumor size (cm)	2.5 ± 0.5	1.9 ± 0.8	<0.001
Tumor location (n,%)			0.853
Upper outer quadrant	17 (54.8%)	126 (48.6%)	
Lower outer quadrant	9 (29.0%)	96 (37.1%)	
Lower inner quadrant	3 (9.7%)	23 (8.9%)	
Upper inner quadrant	2 (6.5%)	14 (5.4%)	
Pre-operative MRI (n,%)	4 (12.9%)	20 (7.7%)	0.322
False negative IFSM (n,%)	8 (25.8%)	20 (7.7%)	0.001
Clinical stage (n,%)			0.693
0	8 (25.8%)	40 (15.4%)	
IA	6 (19.4%)	59 (38.2%)	
IB	7 (22.6%)	69 (26.6%)	
IIA	6 (19.4%)	58 (22.4%)	
IIB	4 (12.9%)	33 (12.7%)	
Histological type (n,%)			<0.001
DCIS component	13 (41.9%)	31 (12.0%)	
Invasive only	18 (58.1%)	228 (88.0%)	
Histological grade (n,%)			0.928
Grade 1	4 (12.9%)	34 (13.1%)	
Grade 2	17 (54.8%)	150 (57.9%)	
Grade 3	10 (32.3%)	75 (29.0%)	
Clinical subtype (n,%)			0.641
Luminal A	10 (32.3%)	118 (45.6%)	
Luminal B	7 (22.6%)	56 (21.6%)	
HER2-positive	5 (16.1%)	27 (10.4%)	
Triple negative	4 (12.9%)	28 (10.8%)	
Ki-67 (n,%)			0.535
Ki-67≥20%	14 (45.2%)	102 (39.4%)	
Ki-67<20%	17 (54.8%)	157 (60.6%)	
Lymphovascular invasion (n,%)	13 (41.9%)	104 (40.2%)	0.849
Axillary lymph node metastasis (n,%)	11 (35.5%)	100 (38.6%)	0.735

CMT, conversion to mastectomy; BCS, breast conserving surgery; BMI, body mass index; MRI, magnetic resonance imaging; IFSM, intra-operative frozen section margin; DCIS, ductal carcinoma *in situ*; HER2, human epidermal growth factor receptor;.

**Figure 2:**
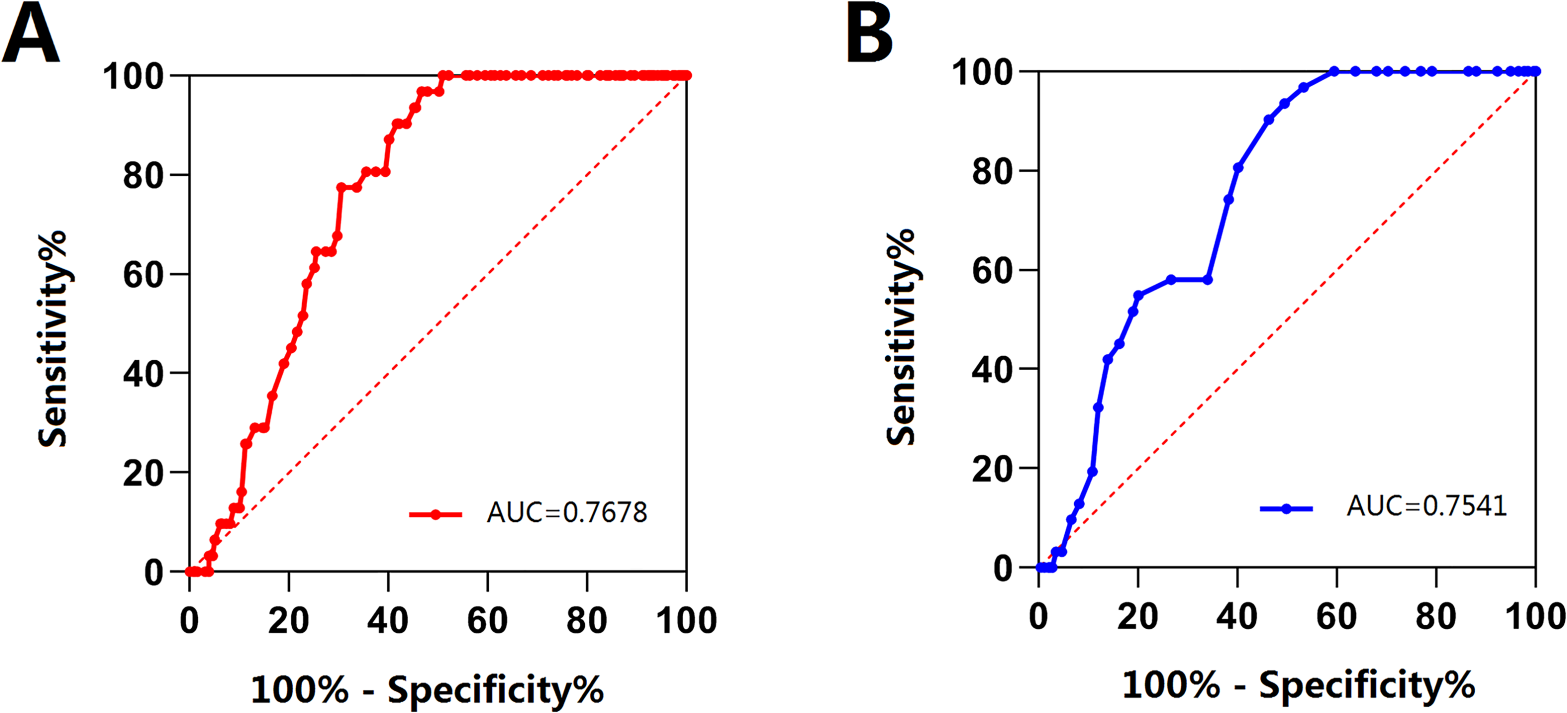
ROC curves of logistic model. ROC, receiver operating characteristic. **(A)** BMI (body mass index); **(B)** Tumor size.

Five parameters demonstrated significant association with CMT in univariable analysis: body mass index (BMI), multifocality, tumor size, false-negative intraoperative frozen section margin, and ductal carcinoma *in situ* (DCIS) component (*P* < 0.05) (**Table 2**). ROC curves were generated for BMI and tumor size, yielding area under the curve (AUC) values of 0.7678 and 0.7541, respectively (**Figure 2**). Variables with *P* < 0.10 in univariable analysis were entered into multivariable logistic regression, which confirmed low BMI (odds ratio [OR] 4.611, 95% confidence interval [CI] 1.875–11.339, *P* = 0.001), multifocality (OR 4.863, 95% CI 1.213–19.494, *P* = 0.026), larger tumor size (OR 3.197, 95% CI 1.670–6.119, *P* < 0.001), DCIS component (OR 5.308, 95% CI 1.126–25.011, *P* = 0.035) as independent risk factors for CMT (**Table 3**).

**Table 2:** Univariable logistic regression for risk factors for conversion to mastectomy.

Variables	B	S.E.	Wals	OR	95%CI	*P* value
BMI	1.621	0.408	15.800	5.057	2.274-11.244	<0.001
Multifocality	1.597	0.542	8.677	4.940	1.707-14.299	0.003
Tumor size	1.121	0.271	17.109	3.068	1.804-5.219	<0.001
False negative IFSM	1.425	0.472	9.116	4.157	1.648-10.481	0.003
DCIS component	1.536	0.415	13.655	4.654	2.058-10.487	<0.001

BMI, body mass index; IFSM, intra-operative frozen section margin; DCIS, ductal carcinoma *in situ*; BC, breast cancer.

**Table 3:** Multivariable logistic regression for risk factors for conversion to mastectomy.

Variables	B	S.E.	Wals	OR	95%CI	*P* value
BMI	1.528	0.459	11.085	4.611	1.875-11.339	0.001
Multifocality	1.582	0.708	4.986	4.863	1.213-19.494	0.026
Tumor size	1.162	0.331	12.304	3.197	1.670-6.119	<0.001
False negative IFSM	0.895	0.619	2.090	2.448	0.727-8.240	0.148
DCIS component	1.669	0.791	4.454	5.308	1.126-25.011	0.035

BMI, body mass index; IFSM, intra-operative frozen section margin; DCIS, ductal carcinoma *in situ*; BC, breast cancer.

## 4 Discussion

BCS has been established for over half a century as a safe locoregional treatment for early-stage BC. Compared with mastectomy, BCS does not increase the risk of recurrence, and BCS with radiotherapy offers superior overall survival to mastectomy (12–15). Furthermore, BCS improves psychological well-being, cosmetic results, and decreases surgical morbidity (13, 16). The primary objectives of BCS are optimal cosmesis, complete tumor excision, and achievement of negative surgical margins (7). Positive margins are associated with an increased risk of local recurrence (17, 18), making disease-free resection margins a critical factor in reducing recurrence and improving overall survival. However, BCS tends to result in more positive margins, requiring re-excision. When BCS is not feasible or residual disease is extensive, mastectomy may be required. Re-excision requiring CMT after positive margins is associated with greater patient distress, unfavorable aesthetic outcomes, higher morbidity, complications, and increased healthcare costs (19). Therefore, accurate preoperative prediction of positive resection margins would enhance patient counseling and potentially decrease the need for mastectomy following re-excision.

Approximately 20-40% of patients receiving BCS require secondary surgery due to postoperative positive margins, according to the literature (8–10). In the present study, the implementation of frozen section analysis for cavity-shave margins was associated with a re-excision rate of only 3.1%, which is significantly lower than previously reported rates.

Low BMI was found to be a risk factor for CMT in this study. Generally, patients with low BMI have smaller breast volumes and limited subcutaneous fat or glandular tissue. Even minor resection leads to noticeable asymmetry or contour deformities, complicating breast reshaping following tumor excision. Furthermore, when the tumor size is relatively large compared with the breast, achieving negative margins necessitates the removal of a significant portion of breast tissue. Traditionally, loss of 20-50% of breast volume increases the risk of poor cosmesis and may necessitate mastectomy (20).

This study demonstrated that multifocal tumors were associated with positive resection margins, consistent with previous studies (21–23). Furthermore, multifocal tumors were associated with an increased rate of CMT. While the primary goal of BCS is oncological safety, cosmetic outcomes are also important. Removing a large volume of tissue to encompass all multifocal diseases can lead to poor cosmetic results, sometimes surpassing the purpose of breast conservation. However, a less aggressive approach aimed at preserving breast shape may inadvertently result in positive margins, necessitating subsequent CMT.

This study demonstrated that patients with DCIS components were more likely to have positive margins and undergo mastectomy. The underlying factors are as follows: the true extent of DCIS can be difficult to estimate preoperatively, as nonpalpable DCIS may extend beyond the detectable tumor area, potentially contributing to positive margins (24). Additionally, DCIS is often multifocal, meaning that positive surgical margins frequently necessitate re-excision or CMT. In this study, DCIS lesions less than 2 mm from the margin were not classified as positive margins. First, margins wider than 2 mm have not been shown to significantly reduce local recurrence in women receiving adjuvant whole-breast radiation therapy, allowing surgeons to avoid excessively large resections while achieving optimal outcomes (25). Second, cavity-shave margins were used instead of the “no ink on tumor” approach to assess margin status, which can complicate precise measurement of margin width.

Several studies have confirmed that larger tumor size is an independent risk factor for positive surgical margins and the presence of residual tumor (22, 26). This study further identified larger tumor size as a significant predictor for CMT using multivariable analysis. This association may be attributed to two factors. First, larger tumors may be incompletely excised due to the potential underestimation of their true dimensions and location. Second, achieving a balance between adequate oncologic resection and satisfactory cosmetic results in patients undergoing BCS presents an inherent technical challenge.

The decision to perform a BCS or mastectomy for early BC is influenced by multiple clinical variables. Our findings highlight the importance of carefully considering these risk factors when evaluating patient suitability for BCS.

This study has some limitations. First, its retrospective design and relatively small sample size may limit the generalizability of the findings. Although the observed associations reached statistical significance, the wide confidence intervals around the effect estimates indicate limited precision. This is largely attributable to the relatively low number of events during the study period. Future studies with larger sample sizes are warranted to confirm our findings. Second, the postoperative “no ink on tumor” principle was not applied for margin evaluation. Instead, cavity-shave margins were used for intraoperative frozen section analysis, a practice adopted in most hospitals in China. While this approach reduces the re-excision rate, it may impair the accurate evaluation of positive margins. Third, some variables that may affect surgical planning were not investigated, such as tumor-to-breast ratio, invasive lobular cancer, and adoption of oncoplastic surgical techniques. Finally, some non-clinical variables that could influence the risk of CMT during BCS were not included in this study.

## 5 Conclusions

In patients with early BC undergoing BCS, low BMI, multifocality, larger tumor size, and the presence of a DCIS component were identified as independent risk factors for CMT due to positive margins. Although these results should be interpreted with caution because of study limitations, recognition of these preoperative risk factors can guide surgical planning and decision-making to optimize oncologic and cosmetic outcomes.

## Data Availability

The raw data supporting the conclusions of this article will be made available by the authors, without undue reservation.
